# IFSO Worldwide Survey 2020–2021: Current Trends for Bariatric and Metabolic Procedures

**DOI:** 10.1007/s11695-024-07118-3

**Published:** 2024-03-04

**Authors:** Luigi Angrisani, Antonella Santonicola, Paola Iovino, Rossella Palma, Lilian Kow, Gerhard Prager, Almino Ramos, Scott Shikora, the Collaborative Study Group for the IFSO Worldwide Survey, Felipe Fiolo, Jorge L. Harraca, Jeff Hamdorf, Felix Langer, Philipp Beckerhinn, Taryel Omerov, Bruno Dillemans, Erick Hassan Bakry Rodriguez, Fabio Viegas, Konstantin Grozdev, Mehran Anvari, Stephen Glazer, Camilo Boza Wilson, Francisco Pacheco Bastidas, Wah Yang, Cunchuan Wang, Luis Ernesto Lopez, Marios Pedonomou, Martin Hruby, Martin Haluzik, Ricardo Domingo, Pablo Garcia, Daniel Guerron, Khaled Gawdat, Alaa Abbas, Tatiana Velarde, Paulina Salminen, Vincent Frering, Dieter Birk, George Skroubis, Harry Pappis, Fernando Montufar, Simon Wong, Praveen Raj, Alireza Khalaj, Ramiz Al Mukhtar, Nasser Sakran, Marco Antonio Zappa, Shinichi Okazumi, Hisahiro Matsubara, Ashraf Haddad, Sami Salem Ahmad, Oral Ospanov, Dong Jin Kim, Sang Kuon Lee, Almantas Maleckas, Nik Ritza Kosai, José G. Rodríguez Villarreal, Simon Nienhuijs, Marloes Emous, Jon Kristinsson, Ricardo Olmedo Bareiro, Wieslaw Tarnowski, Jorge Santos, Nesreen Mahmoud Khidir, Catalin Copaescu, Bekkhan Khatsiev, Aayed Alqahtani, Kim Guowei, Chun Hai Tan, Tadeja Pintar, Tess van der Merwe, Esteban Martin Antona, Andrés Sánchez Pernaute, Johan Ottosson, Torsten Olbers, Felix Bauknecht, Marco Bueter, Weu Wang, Halit Eren Taskin, Mustafa Sahin, Basim Alkhafaji, Abdulwahid Alwahidi, Vinod Menon, Sergio Sauto, Benjamin Clapp, Teresa LaMasters, Luis Rafael Level Cordova

**Affiliations:** 1 Public Health Department - School of Medicine, “Federico II” University of Naples, Naples, Italy; 2 Gastrointestinal Unit, Department of Medicine, Surgery and Dentistry, University of Salerno, Salerno, Italy; 3 Department of Surgical Sciences, “Sapienza” University of Rome, Rome, Italy; 4 Flinders University, Adelaide, South Australia; 5 Division of Visceral Surgery, Department of General Surgery, Medical University of Vienna, Vienna, Austria; 6 Gastro-Obeso-Center Institute, Sao Paulo, Brazil; 7 Brigham and Women’s Hospital, Harvard Medical School, Boston, MA USA

**Keywords:** Metabolic and bariatric surgery, COVID-19 pandemic, IFSO chapters

## Abstract

**Purpose:**

This IFSO survey aims to describe the current trends of metabolic and bariatric surgery (MBS) reporting on the number and types of surgical and endoluminal procedures performed in 2020 and 2021, in the world and within each IFSO chapter.

**Methods:**

All national societies belonging to IFSO were asked to complete the survey form. The number and types of procedures performed (surgical and endoluminal interventions) from 2020 to 2021 were documented. A special section focused on the impact of COVID-19, the existence of national protocols for MBS, the use of telemedicine, and any mortality related to MBS. A trend analysis of the data, both worldwide and within each IFSO chapter, was also performed for the period between 2018 and 2021.

**Results:**

Fifty-seven of the 74 (77%) IFSO national societies submitted the survey. Twenty-four of the 57 (42.1%) reported data from their national registries. The total number of surgical and endoluminal procedures performed in 2020 was 507,806 and in 2021 was 598,834. Sleeve gastrectomy (SG) remained the most performed bariatric procedure. Thirty national societies (52%) had regional protocols for MBS during COVID-19, 61.4% supported the use of telemedicine, and only 47.3% collected data on mortality after MBS in 2020. These percentages did not significantly change in 2021 (*p* > 0.05).

**Conclusions:**

The number of MBS markedly decreased worldwide during 2020. Although there was a positive trend in 2021, it did not reach the values obtained before the COVID-19 pandemic. SG continued to be the most performed operation. Adjustable gastric banding (AGB) continues to decrease worldwide.

**Graphical Abstract:**

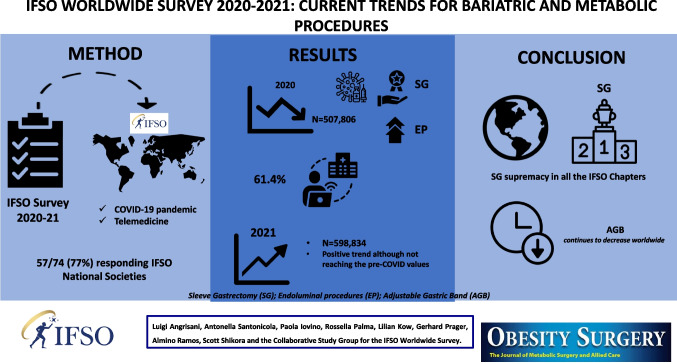

**Supplementary Information:**

The online version contains supplementary material available at 10.1007/s11695-024-07118-3.

## Introduction

The International Federation for the Surgery of Obesity and Metabolic Disorders (IFSO) aims to promote the dissemination of scientific knowledge to inform and orientate bariatric surgeons and integrated health professionals in their daily clinical practices. Since the presidential address of Nicola Scopinaro during the Second Congress of the IFSO in Cancun on October 1997 [1], the ambitious idea of collecting the number and type of operations performed in the national societies belonging to IFSO was introduced resulting in several surveys published [2–7]. The total number of MBS has steadily increased worldwide, and bariatric surgeons have been constantly evolving their opinion regarding the safety and efficacy of the different bariatric procedures over the years.

The last 2018 IFSO survey [7], confirmed that sleeve gastrectomy (SG) was the most performed operation, the decline of adjustable gastric band (AGB), and the increasing popularity of one-anastomosis gastric bypass (OAGB).

This survey aims to describe the number and types of procedures performed in 2020 and 2021, in the world and within each IFSO chapter taking into account the negative impact of the pandemic “coronavirus disease 19” (COVID-19). Elective surgeries were cancelled or delayed to reallocate medical personnel and equipment towards helping the intensive care unit (ICU) capacity. In May 2020, the IFSO position statement [8] recommended that elective surgical and endoscopic cases for MBS should be postponed during the pandemic and that clinic and hospital follow-up visits should be delayed and replaced by video consultations or other types of telemedicine. Consequently, a special section was introduced in this survey regarding the existence of national COVID protocols for bariatric and metabolic procedures, the use of telemedicine, and the collection of data on mortality after bariatric and metabolic procedures during the pandemic.

## Methods

The 2020–2021 IFSO survey form was emailed by the IFSO secretariat to all the national IFSO societies in March 2022. The form is mainly required to report the type of operations and number of primary and revisional procedures performed.

Regarding the data source, it was specifically asked if they were provided by national registry, national survey, expert opinion, or by other means. It has to be considered that 72 IFSO national societies are spread over five earth continents with large diversity in terms of Health Service System, economic resources, level of organization, and methods of data collection. In case of the absence of official answer after repeated emails, direct telephone contact was used with the National Society President and well-known senior surgeons leaders in the field. Only in case of a total lack of recorded data system, expert opinion was stimulated to provide the required information.

**Fig. 1:**
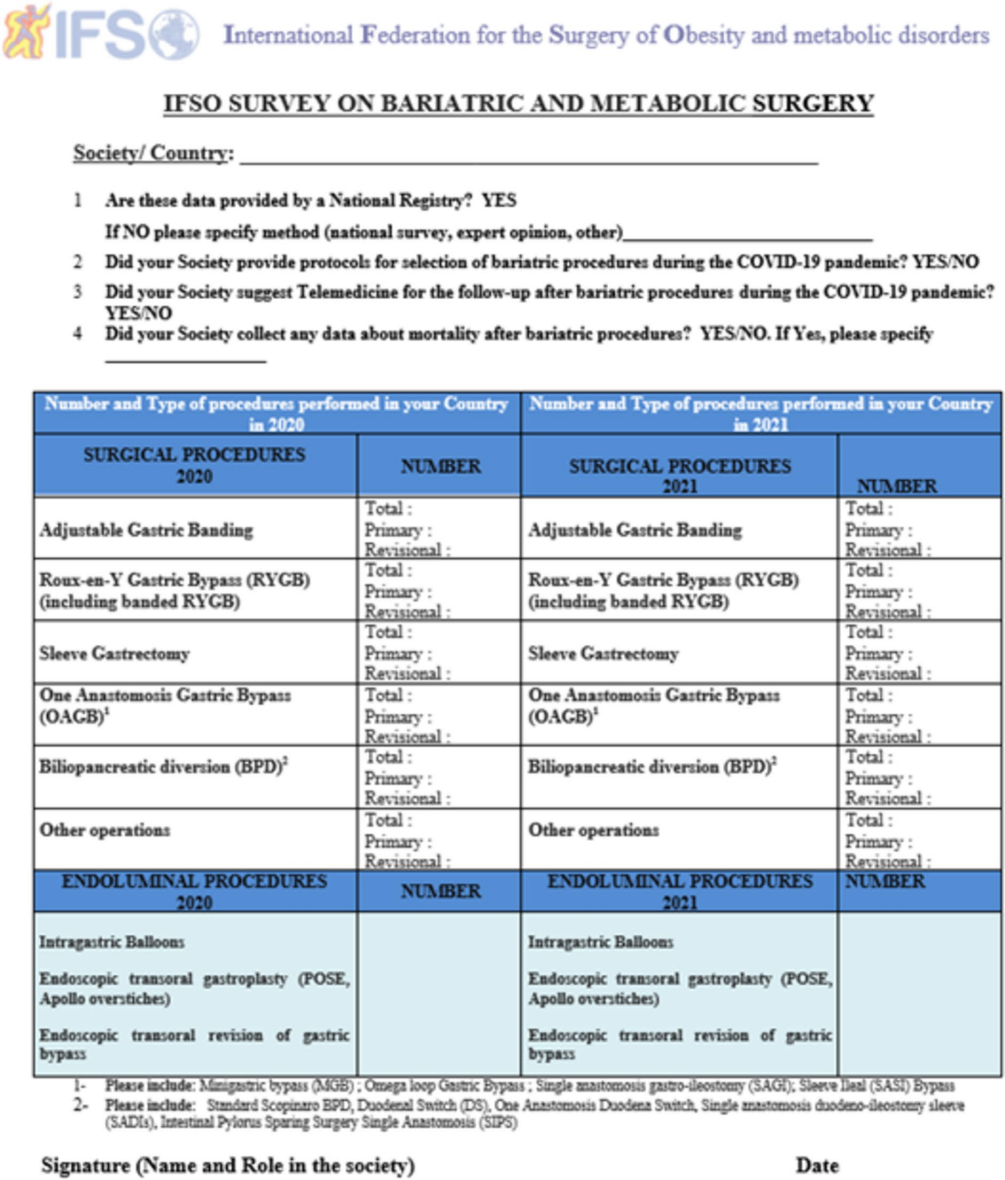
The IFSO survey form 2020–2021

Part of this survey was also dedicated to COVID-19: the adoption of national protocols for procedure selection, the use of telemedicine, and data on mortality after MBS during the pandemic (Fig. 1). The data from Canada came only from Ontario.

The results of the 2020–2021 IFSO survey were compared to the last 2018 published survey.

The obtained data were expressed as absolute and relative frequencies. The relative frequency was calculated by dividing the absolute frequency by the total number of values for the variable.

## Results

The IFSO survey form 2020–2021 (Table 1) was sent to 74 IFSO national societies. Fifty-seven (77%) of the national IFSO societies completed and submitted the form.

**Table 1:** The number and the type of operations worldwide in 2018, 2020, and 2021

	2018	2020	2021
Sleeve gastrectomy (SG)	386,096	304,352	351,689
Roux-en-Y gastric bypass (RYGB)	203,769	133,007	159,543
One anastomosis gastric bypass (OAGB)	46,406	29,117	46,113
Biliopancreatic diversion (BPD)	6506	6896	7973
Adjustable gastric banding (AGB)	9757	6116	5010
Other surgical operations	14,346	13,949	13,238
Intragastric balloons	27,780	11,492	12,421
Other endoluminal procedures	1531	2877	2707
Total	**696,191**	**507,806**	**604,099**

Costa Rica, Egypt, Honduras, Hungary, Iceland, Indonesia, Kuwait, Lebanon, Pakistan, Panama, Perù, the Philippines, Serbia, Sri Lanka, Thailand, Ukraine, and Uzbekistan did not respond.

Twenty-four (42.1%) IFSO societies reported data from their national registries.

The total number of surgical and endoluminal procedures performed in the world in 2020 was 507,806 and in 2021 was 598,834. Considering the last IFSO survey report [7], the number of bariatric and metabolic procedures decreased, as reported in Table 1.

**Fig. 2:**
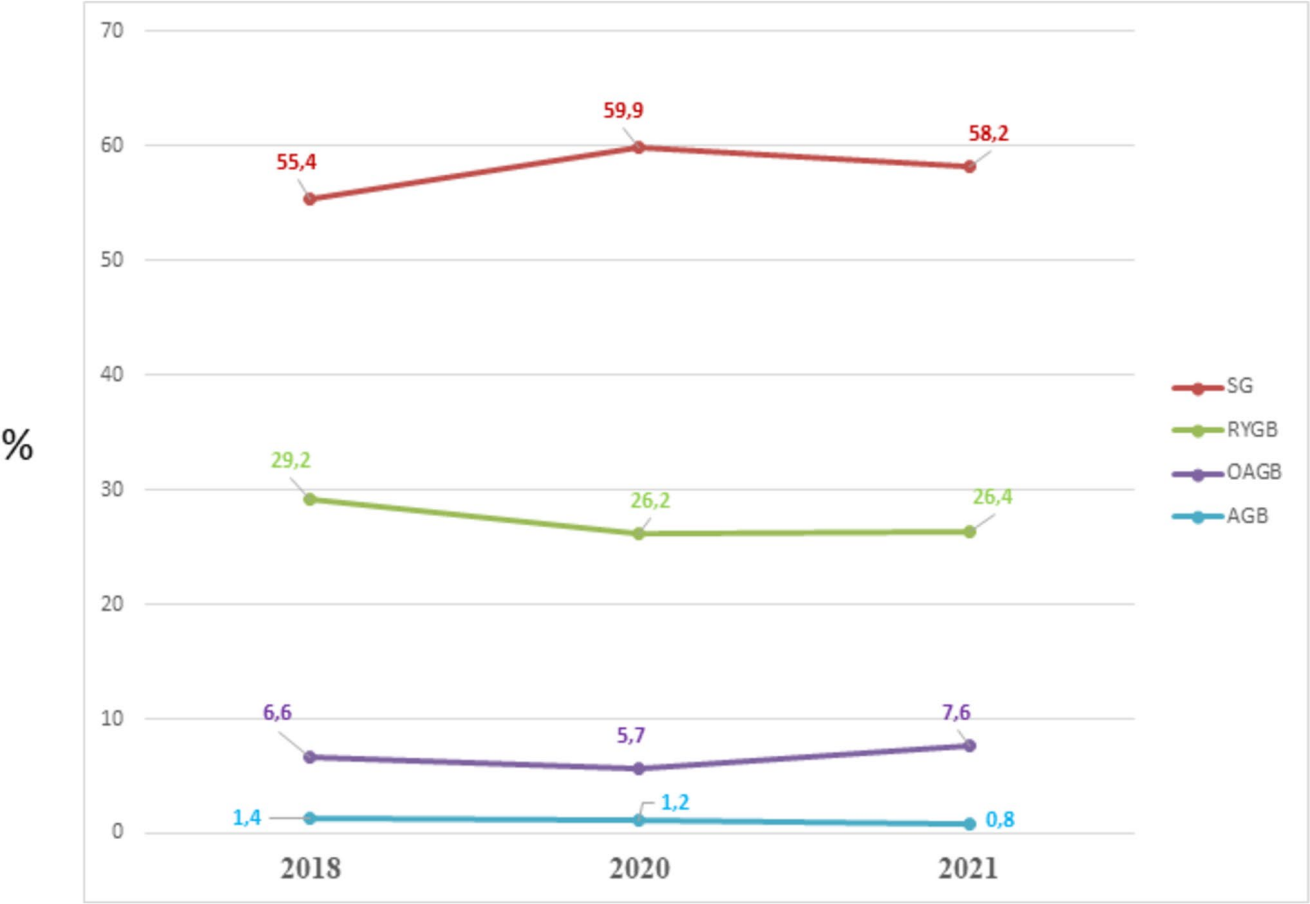
The worldwide trend from 2018 to 2021 of the main bariatric/metabolic procedures

Figure 2 demonstrates the worldwide trend in the percentage of the main surgical bariatric and metabolic operations from 2018 to 2021, which continued to increase though only slightly over time and confirmed that the SG was the most commonly performed MBS in the world.

Thirty (52%) of the IFSO national societies had national protocols for bariatric procedures, 35 (61.4%) of the national societies used telemedicine for patient follow-up after a MBS, and 27 (47.3%) societies collected data on mortality after MBS. These percentages did not significantly change in 2021 (*p* > 0.05).

There was not any significant difference among the IFSO chapters in the percentages of adoption of national protocols for bariatric and metabolic procedures both in 2020 and 2021. The use of telemedicine in 2021 was higher in LAC and NAC compared to the other IFSO societies (*p* = 0.045).

## Asia Pacific Chapter (APC)

The total number of surgical and endoluminal procedures performed in the APC in 2020 was 63,343 and in 2021 was 76,635. In the last IFSO survey report [7], 70,573 bariatric procedures in APC were reported in 2018. While MBS performed in the APC, declined in 2020, it increased in 2021 (Table 2).

**Table 2:** The number and the type of operations performed in the APC in 2018, 2020, and 2021

	2018	2020	2021
Sleeve gastrectomy (SG)	38,160	40,408	52,398
Roux-en-Y gastric bypass (RYGB)	9488	8712	9386
One anastomosis gastric bypass (OAGB)	14,164	9127	11,450
Biliopancreatic diversion (BPD)	1103	521	639
Adjustable gastric banding (AGB)	1297	371	224
Other surgical operations	2807	2924	1109
Intragastric balloons	3359	980	1109
Other endoluminal procedures	195	300	320
Total	**70,573**	**63,343**	**76,635**

**Fig. 3:**
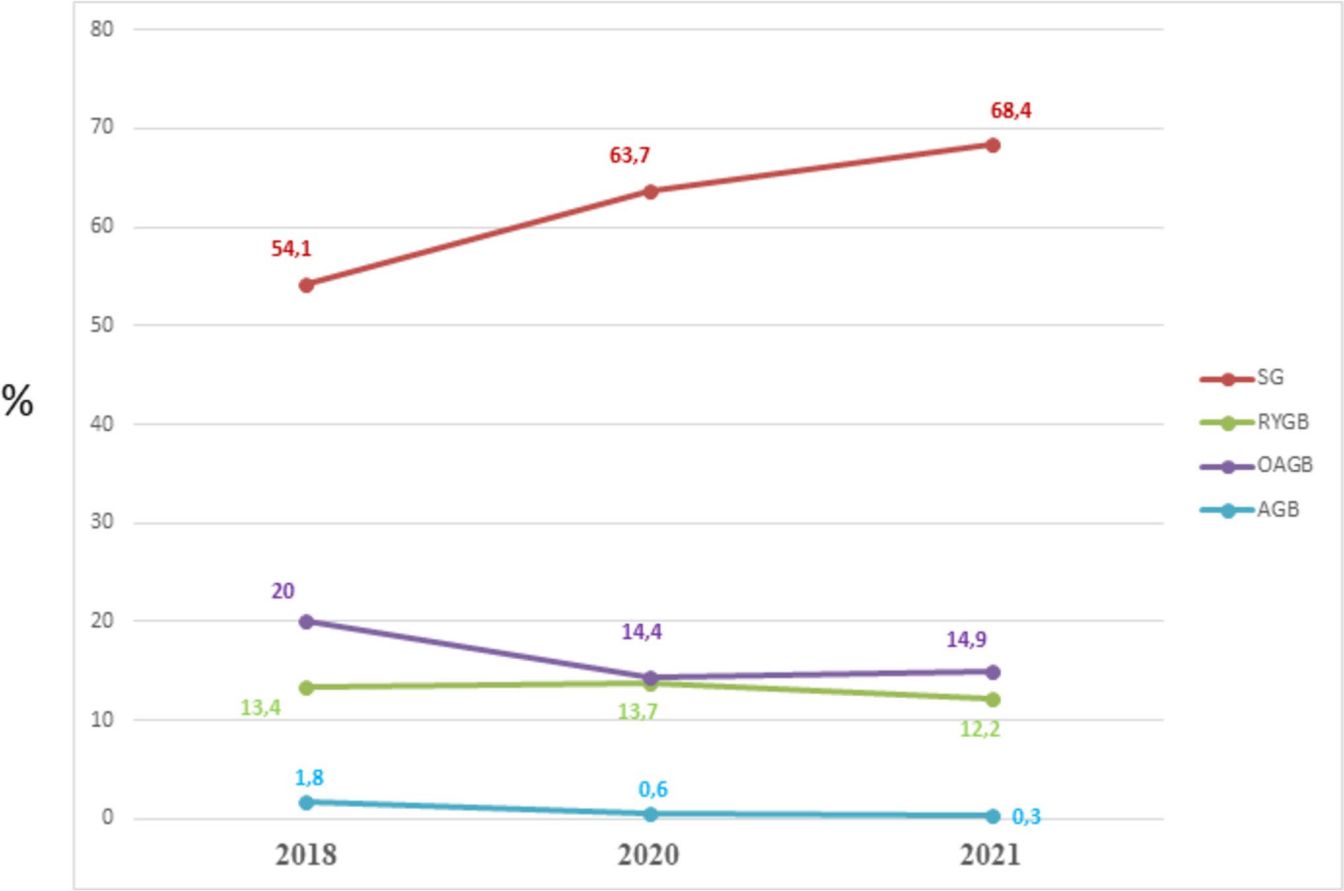
The trend in the percentage of the main surgical bariatric operations performed in the APC from 2018 to 2021

Figure 3 demonstrates the trend in the percentage of the main MBS performed in the APC from 2018 to 2021. There was a further increase of SG in 2021 which now represented about 70% of all the procedures performed. The OAGB remained the second most performed MBS, although it had a slight decrease. The popularity of the AGB continued to decrease and its use was essentially abandoned. This finding is of special interest because in the APC the percentage of AGB was high for a long time.

The IFSO APC societies that had national protocols for MBS, the use of telemedicine, and the data collection about mortality after MBS (during the year 2020 and 2021) were reported in the Supplementary Fig. 1S.

## European Chapter (EC)

The total number of surgical and endoluminal procedures performed in the EC in 2020 was 136,584 and in 2021 was 178,471. Considering the last IFSO survey report [7], the number of MBS performed in 2020 and 2021 was lower than in 2018, as reported in Table 3.

**Table 3:** The number and the type of operations performed in the EC in 2018, 2020, and 2021

	2018	2020	2021
Sleeve gastrectomy (SG)	104,052	63,666	79,499
Roux-en-Y gastric bypass (RYGB)	48,872	42,918	53,918
One anastomosis gastric bypass (OAGB)	21,962	15,633	29,475
Biliopancreatic diversion (BPD)	1322	835	1245
Adjustable gastric banding (AGB)	4592	3013	2942
Other surgical operations	4110	7281	5313
Intragastric balloons	4829	2900	5301
Other endoluminal procedures	613	338	778
Total	**190,352**	**136,584**	**183,736**

**Fig. 4:**
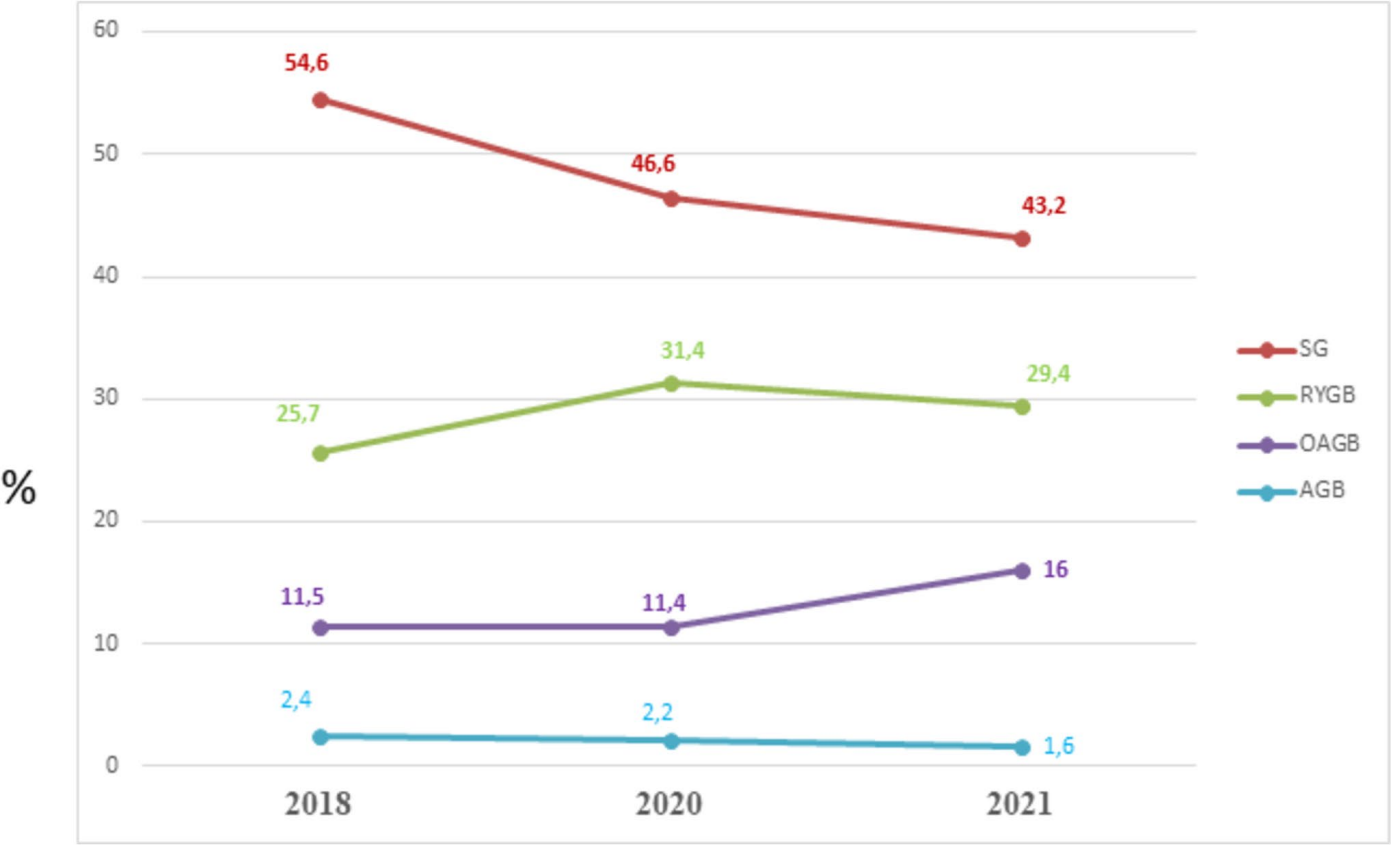
The trend in the percentage of the main surgical bariatric operations performed in the EC from 2018 to 2021

Figure 4 demonstrates the trend in the percentage of the main MBS performed in the EC from 2018 to 2021. There was a decrease in SG, which remained the most commonly performed procedure, and a slight increase in OAGB. RYGB and AGB were stable over time.

The IFSO EC societies that had national protocols for bariatric and metabolic procedures, the use of telemedicine, and the data collection on mortality after MBS during 2020 and 2021 were reported in the Supplementary Fig. 2S.

## Latin America Chapter (LAC)

The total number of surgical and endoluminal procedures performed in the LAC in 2020 was 82,523 and in 2021 was 104,239. Considering the last IFSO survey report [7], the number of MBS performed in 2020 and 2021 was lower than in 2018, as reported in Table 4.

**Table 4:** The number and the type of operations performed in the LAC in 2018, 2020, and 2021

	2018	2020	2021
Sleeve gastrectomy (SG)	56,844	39,515	46,067
Roux-en-Y gastric bypass (RYGB)	94,146	36,004	44,177
One anastomosis gastric bypass (OAGB)	4044	857	1898
Biliopancreatic diversion (BPD)	1138	1436	1795
Adjustable gastric banding (AGB)	578	83	707
Other surgical operations	810	618	4439
Intragastric balloons	13,034	3778	4436
Other endoluminal procedures	555	232	720
Total	**171,149**	**82,523**	**104,239**

**Fig. 5:**
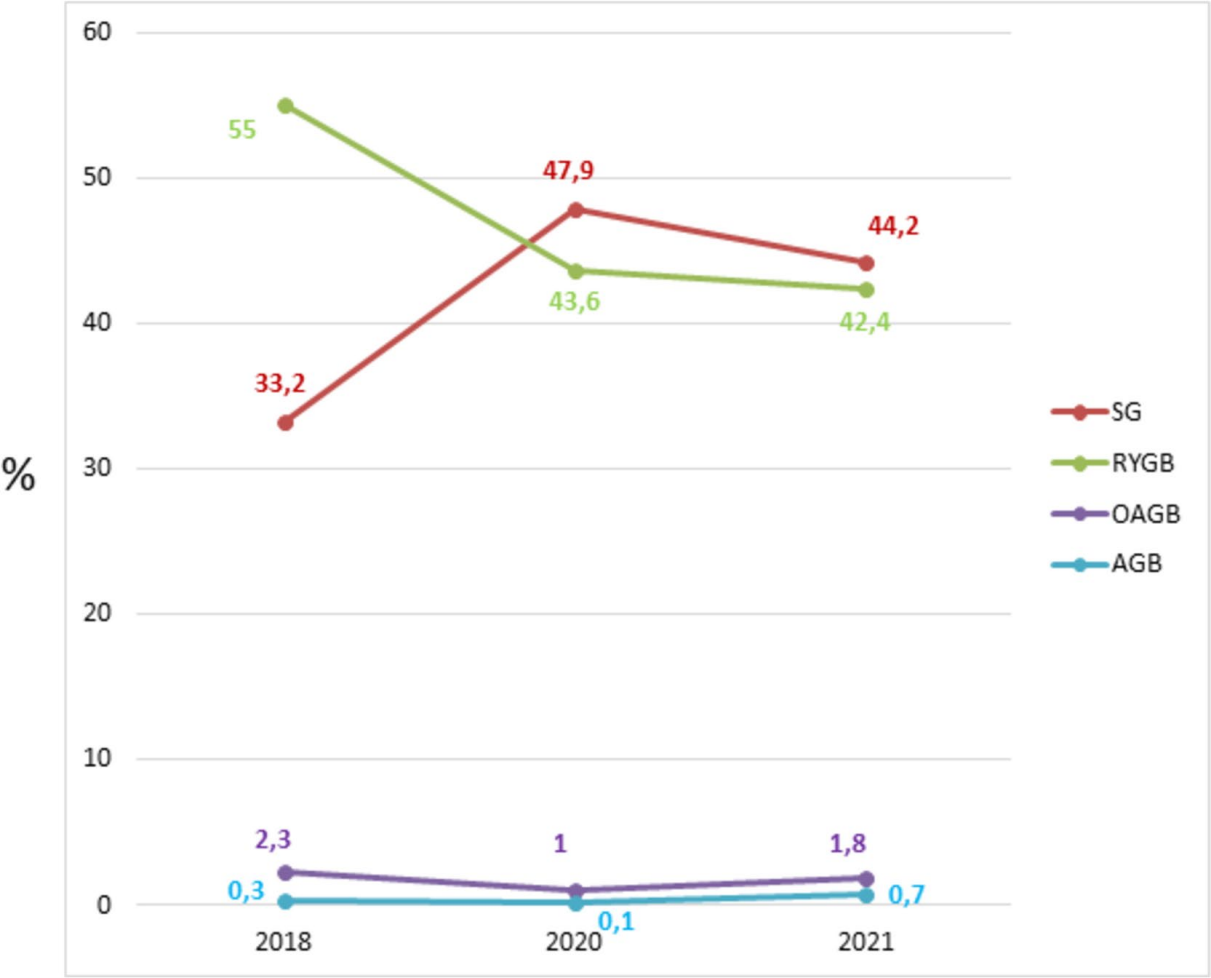
The trend in the percentage of the main surgical bariatric operations performed in the LAC from 2018 to 2021

Figure 5 demonstrates the trend in the percentage of the main surgical bariatric operations performed in the LAC from 2018 to 2021. There was a remarkable increase of SG, which in 2020 overtook the RYGB and became, for the first time in LAC, the most performed procedure. OAGB and AGB were stable and represented a small part of the surgical interventions.

The LAC IFSO societies that used national protocols for MBS, the use of telemedicine, and the data collection on mortality after MBS during 2020 and 2021 were reported in the Supplementary Fig. 3S.

## Middle East and North African Chapter (MENAC)

The total number of surgical and endoluminal procedures performed in the MENAC in 2020 was 46,272 and in 2021 was 52,918. Considering the last IFSO survey report [7], the number of MBS performed, both in 2020 and 2021, was higher than in 2018, as reported in Table 5.

**Table 5:** The number and the type of operations performed in the MENAC in 2018, 2020, and 2021

	2018	2020	2021
Sleeve gastrectomy (SG)	27,579	38,244	44,034
Roux-en-Y gastric bypass (RYGB)	4185	2140	2357
One anastomosis gastric bypass (OAGB)	6236	2162	2346
Biliopancreatic diversion (BPD)	455	25	26
Adjustable gastric banding (AGB)	510	256	174
Other surgical operations	772	1904	1947
Intragastric balloons	1516	1034	1145
Other endoluminal procedures	168	507	889
Total	**41,421**	**46,272**	**52,918**

**Fig. 6:**
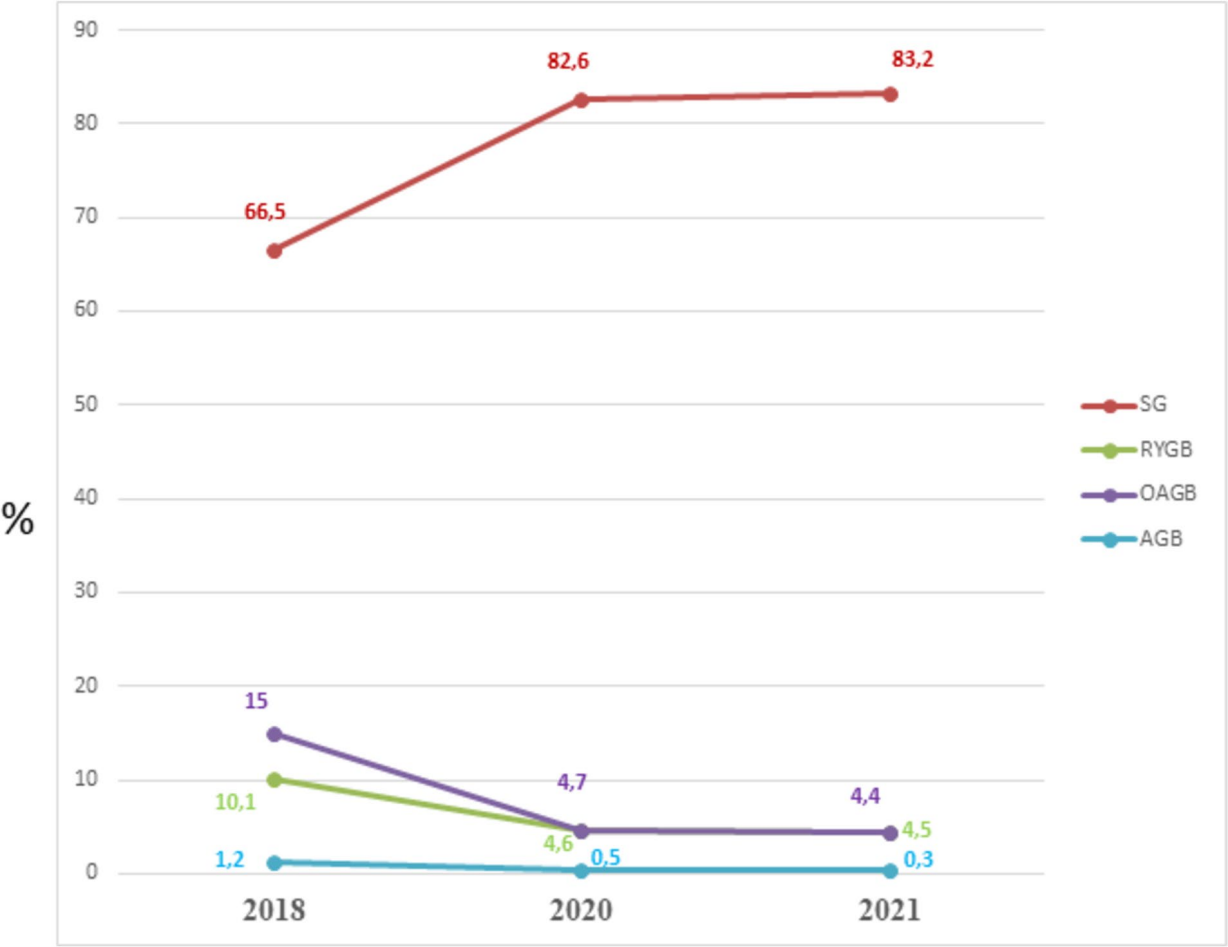
The trend in the percentage of the main surgical bariatric operations performed in the MENAC from 2018 to 2021

Figure 6 demonstrates the trend in the percentage of the main surgical bariatric operations performed in the MENAC from 2018 to 2021. There was a remarkable increase of SG, which represented more than 80% of bariatric interventions. Both OAGB and RYGB decreased, and AGB was abandoned.

The MENAC IFSO societies that used national protocols for MBS, the use of telemedicine, and the data collection on mortality after MBS during 2020 and 2021 were reported in the Supplementary Fig. 4S.

## North America Chapter (NAC)

The total number of surgical and endoluminal procedures performed in the NAC in 2020 was 179,083 and in 2021 was 186,568. Considering the last IFSO survey report [7], the number of MBS performed in 2020 and 2021 was lower than in 2018, as reported in Table 6.

**Table 6:** The number and the type of operations performed in the NAC in 2018, 2020, and 2021

	2018	2020	2021
Sleeve gastrectomy (SG)	159,461	122,519	129,691
Roux-en-Y gastric bypass (RYGB)	47,078	43,233	49,705
One anastomosis gastric bypass (OAGB)	n.r	1338	944
Biliopancreatic diversion (BPD)	2488	4079	4408
Adjustable gastric banding (AGB)	2780	2393	960
Other surgical operations	5847	1221	430
Intragastric balloons	5042	2800	430
Other endoluminal procedures	n.r	1500	n.r
Total	**222,696**	**179,083**	**186,568**

**Fig. 7:**
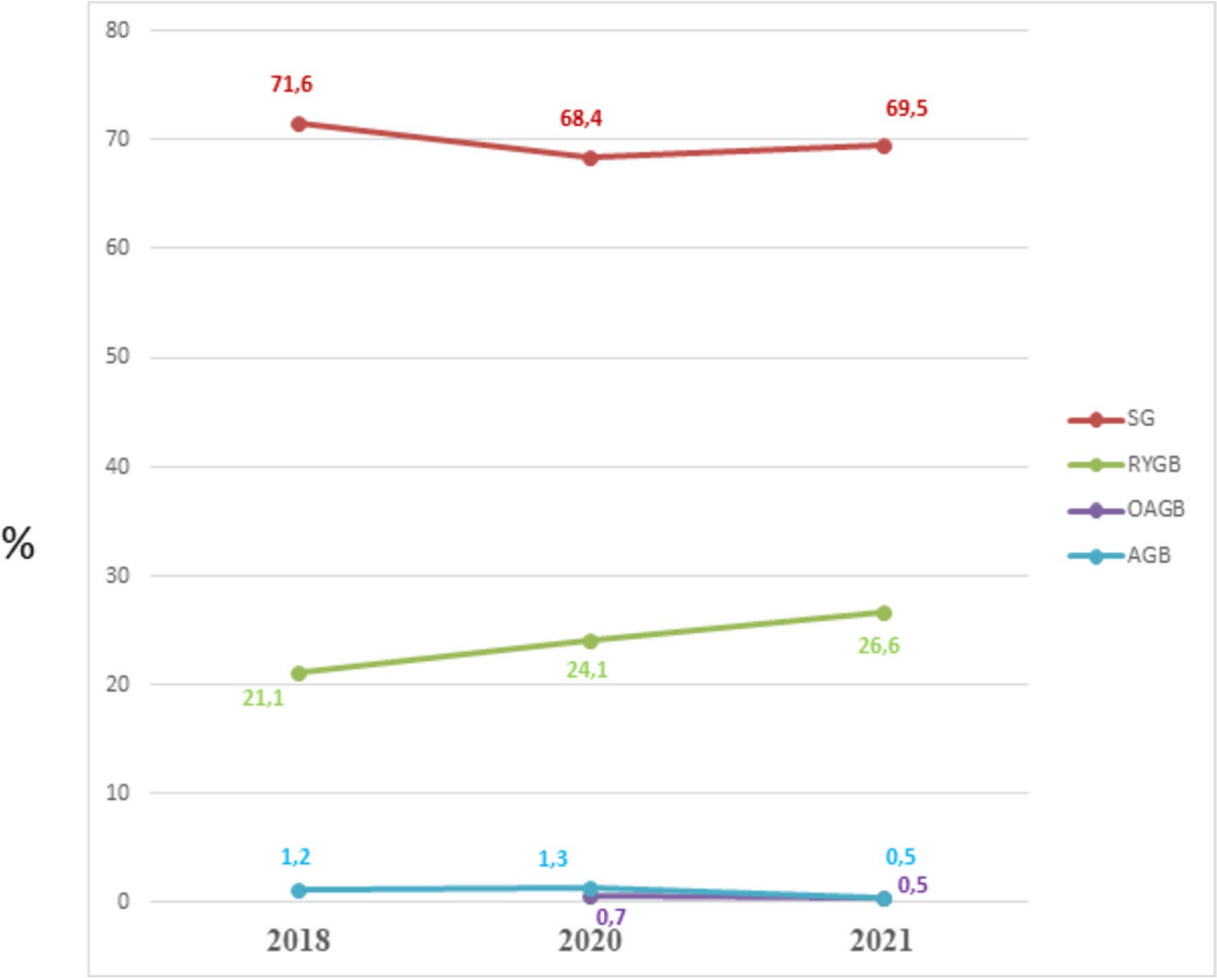
The trend in the percentage of the main surgical bariatric operations performed in the NAC from 2018 to 2021

Figure 7 demonstrates the trend in the percentage of the main surgical bariatric operations performed in the NAC from 2018 to 2021. SG was stable over time representing about 70% of all bariatric interventions. RYGB had a slight increase. From 2020, data about the OAGB were available, but it represented < 1% of the bariatric surgical interventions.

The IFSO NAC societies that used national protocols, the use of telemedicine, and the data collection on mortality as MBS during 2020 and 2021 were reported in the supplementary Fig. 5S.

## Discussion

The IFSO survey which has been repeatedly published over the years, describes the total number of procedures performed worldwide for MBS and the trends analysis based on the surgeons’ preference for one type of operation over another. These data were also provided for each individual IFSO chapter to recognize specific attitudes and trends in different world regions. The results of the IFSO surveys over the years have been a source of relevant and useful information for clinical practice and the scientific community dedicated to the treatment of obesity and metabolic disorders. In fact, this was clearly confirmed by the scientific literature based on the large number of received citations.

Regarding the operation of choice for many surgeons, the supremacy of SG was maintained over the bypass operations worldwide [9] but without a significant growing trend as in the previous survey. Roux-en-Y gastric bypass (RYGB) and one anastomosis gastric bypass (OAGB) remain the most commonly performed operations after SG, with a consistent lead over the other procedures. There was a worldwide increase in endoscopic bariatric procedures such as endoscopic sleeve gastroplasty and the transoral outlet reduction after gastric bypass (from 1531 in 2018 to 2707 in 2021). A possible explanation of these unexpected findings could be the preference of some bariatric centers to adopt less invasive endoscopic procedures rather than standard MBS due to COVID’s coexistence. Certainly, gastroenterologists are increasingly involved in the management of patients with obesity. However, the number of endoscopic procedures reported in this survey is still limited by the fact that only a few gastroenterologists belong to the national bariatric societies and their data are not often officially available.

From 2018 to 2021 the number of AGB implanted was reduced by half.

As expected, the total number of bariatric surgery cases markedly decreased during 2020, with a partial rising trend in 2021. However, it did not reach the values before the COVID-19 pandemic. This should not be interpreted as a true decrease in case volume worldwide but only as a lower response rate to this survey when compared to the IFSO 2018 survey (87.7% vs 77%).

Amongst the non-responders of the IFSO national societies there were Indonesia, the Philippines, Sri Lanka, and Thailand. However, in the published IFSO-APC National Reports 2021[10] these national societies reported to have performed a combined total of only 841 procedures. In addition, for the first time, Ukraine, Egypt, and Peru did not respond at all, probably due to lack of resources in their local healthcare services as a result of catastrophes such as the war and/or the COVID-19 pandemic. Although the worldwide trends of MBS did not significantly change before and after the COVID-19 pandemic, we revealed substantial differences among the IFSO chapters.

In the European and NA chapters, SG remained the most popular operation, although it was stable or had a slight decrease. On the contrary, in AP and MENA chapters, SG had a marked increase in numbers compared to 2018 (+ 14% and + 16%, respectively). Moreover, during the COVID-19 pandemic, SG became, for the first time, the most-performed operation in the LAC, ending the RYGB supremacy in that region. Since 2014, the SG has continued to be the most performed operation, probably due to its already-reported advantages [9]. Furthermore, the global health emergency due to the COVID-19 pandemic, which significantly impacted health services, probably changed the operative strategy of bariatric surgeons worldwide preferring a simpler operation with fewer surgical complications.

RYGB remained the second most commonly performed surgical operation in the world, as well as in European and North American chapters. On the contrary, in AP and MENA chapters, it represented the third most commonly performed procedure, after the OAGB. In 2020, in the LAC, the RYGB lost, for the first time, its supremacy as the most common operation performed. The OAGB was stable globally. It was the third most performed procedure. However, in the European chapter, it had a slight increase (+ 4%). In the AP and MENA chapters, the OAGB decreased and in the LA and NA chapters, it represented only a minority of the performed operations.

This survey also demonstrated an interesting increase in other unconventional operations, totaling 13,238 in 2021. Although the IFSO bariatric and metabolic community is committed to the standardization of surgical procedures through position statements and guidelines, there were a consistent number of procedures that were performed that were not included in the list of officially recognized interventions.

As already reported in the previous IFSO surveys [3–7], the critical limiting factor for the scientific value of this report is the quality of the data and the methodology of collection. Data were obtained from an emailed questionnaire sent to the IFSO national societies but only 42% of them reported numbers extrapolated from the official national registry and about 32% from the National Surveys.

Both these data sources present consistent limitations. Registries undoubtedly produce better quality data [11], but are far from being complete, since many surgeons are reluctant to spend time to insert the required items and often are not willing to share detailed patient information, especially in the case of private practice settings. Registries, in fact, are mostly useful for outcome analysis. The Official National Society Survey does not require specific patient data and generally receives a much wider surgeons’ participation. Therefore, it might be considered more suitable for trend analysis.

Although the methodology is not flawless as previously pointed out, this IFSO survey offers a global overview of MBS performed in the national societies taking also into account the COVID-19 pandemic. The COVID-19 outbreak has dramatically reduced the number of bariatric and metabolic procedures. From 2021, we documented the “recovery” of MBS possibly due to the very rapid knowledge about this new coronavirus and, the subsequent widespread use of vaccinations. These results have enhanced the pivotal role of MBS in the management of patients with obesity and their comorbidities. In November 2020, the IFSO LAC published recommendations for the resumption of elective bariatric metabolic surgery during the COVID-19 pandemic. The pandemic demonstrated that patients with severe obesity had higher mortality due to COVID-19 compared to the general population [10] and that for these patients, MBS must be considered “life-saving” as well as surgical oncology. More recently, a multinational cohort study showed that with appropriate perioperative protocols [12], MBS could be safely performed also during the COVID-19 pandemic.

The use of telemedicine and video consultation deriving from patient management during the pandemic was another very important point reported in this study. Currently, these modern technologies have been rapidly incorporated by the bariatric surgeon’s community for patient management remotely.

In conclusion, this IFSO global survey confirmed that the sleeve gastrectomy is the most performed surgical procedure worldwide. It was not observed for any consistent growth and its trend is probably reaching a plateau. The AGB continues to decrease, and it may be that it will become an operation for historical purpose only. The endoluminal procedures are definitively increasing and current data are largely underestimated because these operations are mostly performed by medical gastroenterologists who are not generally involved in the national bariatric surgical societies.

The volume of MBS cases in the future will continue to increase with some interesting trends towards perhaps less invasive procedures. As such, the IFSO worldwide survey will continue to report on the global trends in years to come. It will continue to be devoted to witness and report on the changing trends of the different MBS procedures. These changing trends over time will reflect on the preference of surgeons globally and the evolution of the current and emerging techniques. Telemedicine and video consultation have officially entered clinical practice for the management of patients with obesity.

### Supplementary Information

Below is the link to the electronic supplementary material.

**Fig. 1S.** The IFSO APC Societies that declared the presence or absence of national protocols for MBS, the use of telemedicine, and the data collection about mortality after MBS during 2020 and 2021. (TIF 71 KB)

**Fig. 2S.** The IFSO EC Societies that declared the presence or absence of national protocols for MBS the use of telemedicine, and the data collection on mortality after bariatric procedures during 2020 and 2021. (TIF 169 KB)

**Fig. 3S.** The LAC IFSO Societies that used national protocols for MBS, the use of telemedicine, and the data collection on mortality after MBS during 2020 and 2021. (TIF 86 KB)

**Fig. 4S.** The IFSO MENAC Societies that used national protocols for MBS , the use of telemedicine, and the data collection on mortality after MBS during 2020 and 2021. (TIF 46 KB)

**Fig. 5S.** The IFSO NAC Societies that uses national protocols for MBS, the use of telemedicine, and the data collection on mortality after MBS during 2020 and 2021. (TIF 26 KB)

## Data Availability

The data that support the findings of this study are available from the corresponding author [L.A.] upon reasonable request.
