# Supplementary figures and images for: IFSO Worldwide Survey 2020–2021: Current Trends for Bariatric and Metabolic Procedures
Source: Obes Surg. 2024 Mar 4;34(4):1075–85. doi: 10.1007/s11695-024-07118-3 (PMC11026210; doi:10.1007/s11695-024-07118-3)

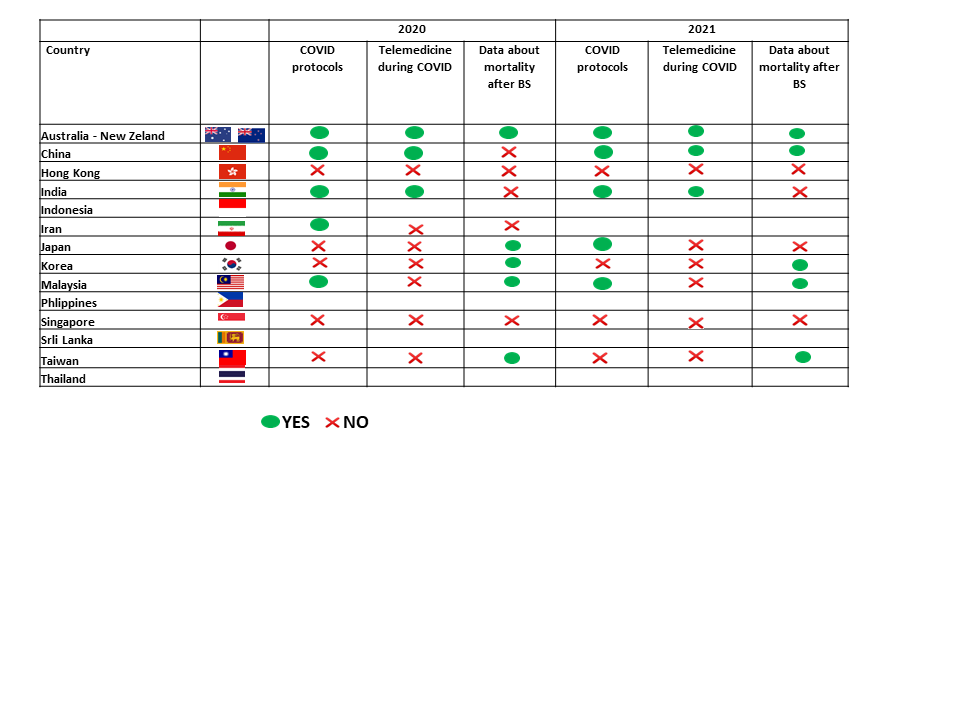

Supplement: Supplementary file 1 — Fig. 1S. The IFSO APC Societies that declared the presence or absence of national protocols for MBS, the use of telemedicine, and the data collection about mortality after MBS during 2020 and 2021. (TIF 71 KB) [file 11695_2024_7118_MOESM1_ESM.tif]

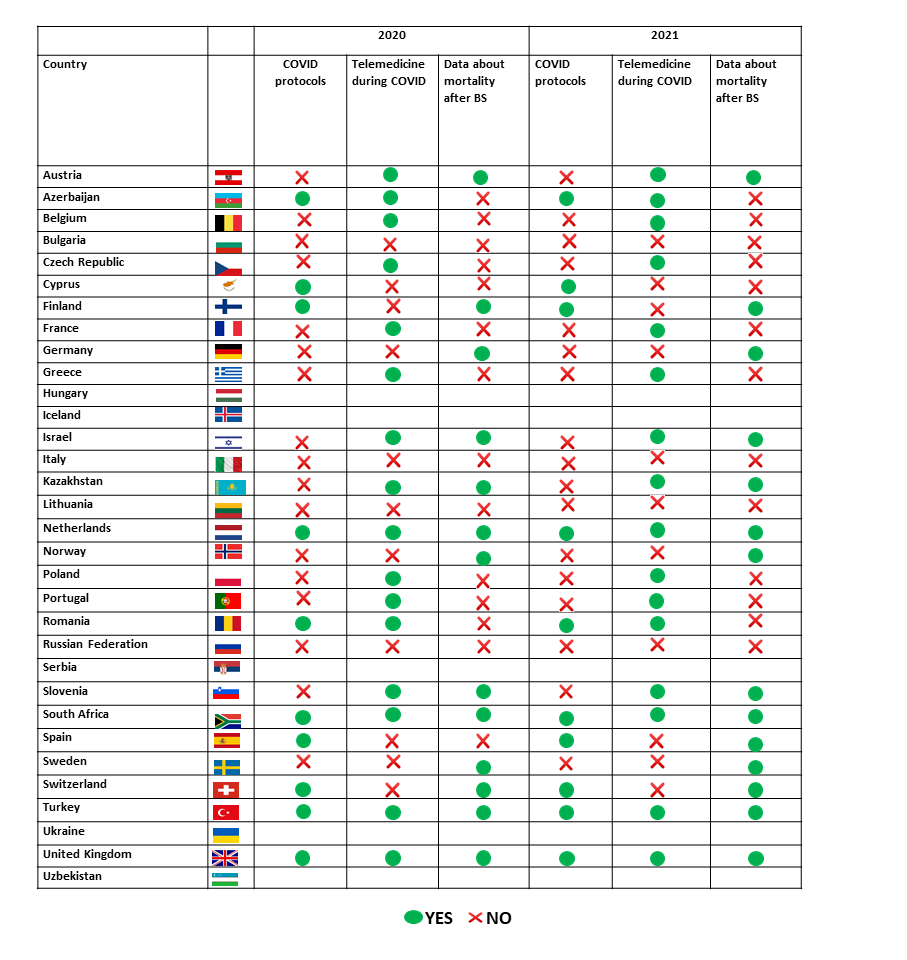

Supplement: Supplementary file 2 — Fig. 2S. The IFSO EC Societies that declared the presence or absence of national protocols for MBS the use of telemedicine, and the data collection on mortality after bariatric procedures during 2020 and 2021. (TIF 169 KB) [file 11695_2024_7118_MOESM2_ESM.tif]

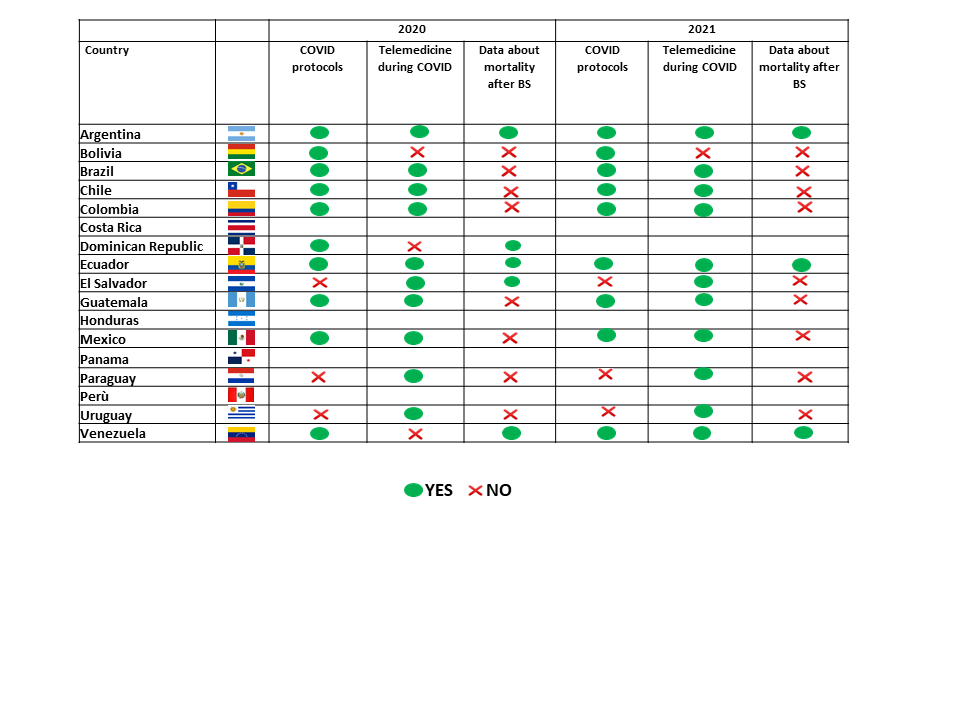

Supplement: Supplementary file 3 — Fig. 3S. The LAC IFSO Societies that used national protocols for MBS, the use of telemedicine, and the data collection on mortality after MBS during 2020 and 2021. (TIF 86 KB) [file 11695_2024_7118_MOESM3_ESM.tif]

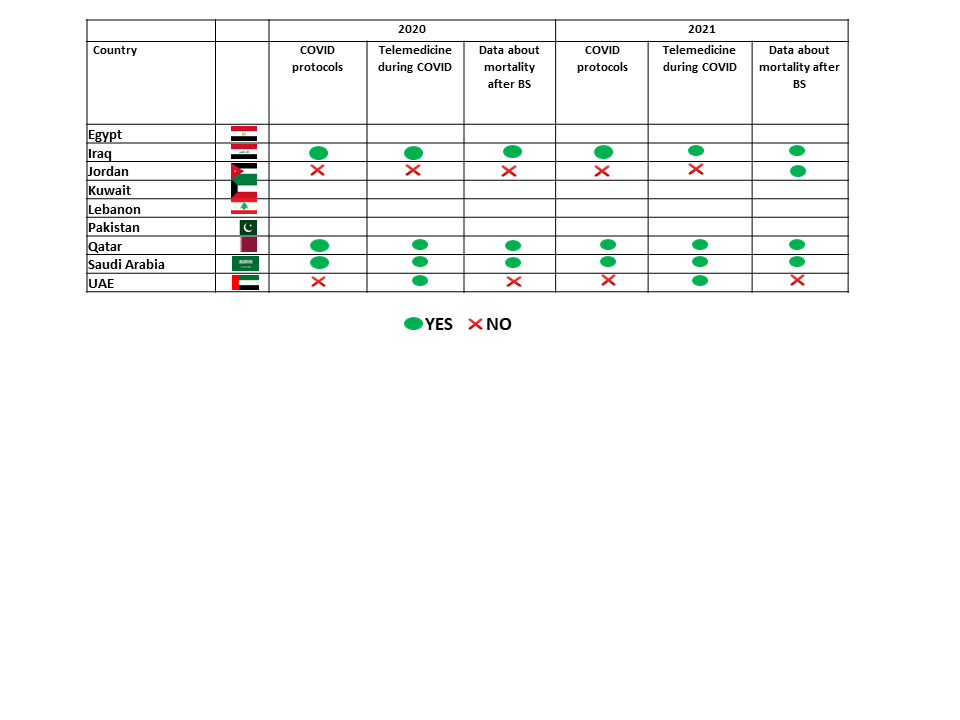

Supplement: Supplementary file 4 — Fig. 4S. The IFSO MENAC Societies that used national protocols for MBS , the use of telemedicine, and the data collection on mortality after MBS during 2020 and 2021. (TIF 46 KB) [file 11695_2024_7118_MOESM4_ESM.tif]

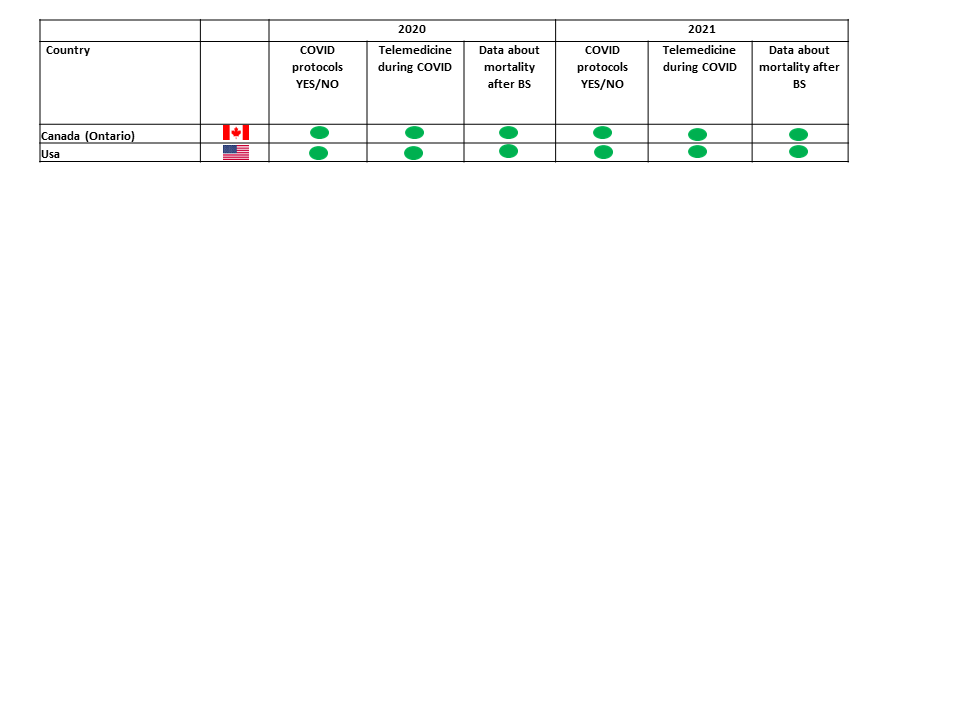

Supplement: Supplementary file 5 — Fig. 5S. The IFSO NAC Societies that uses national protocols for MBS, the use of telemedicine, and the data collection on mortality after MBS during 2020 and 2021. (TIF 26 KB) [file 11695_2024_7118_MOESM5_ESM.tif]
